# Genome-wide identification of the *WRKY* gene family in blueberry (*Vaccinium* spp.) and expression analysis under abiotic stress

**DOI:** 10.3389/fpls.2024.1447749

**Published:** 2024-08-15

**Authors:** Lei Lei, Kun Dong, Siwen Liu, Yadong Li, Guohui Xu, Haiyue Sun

**Affiliations:** ^1^ College of Horticulture, Jilin Agricultural University, Changchun, China; ^2^ College of Life Sciences, Jilin Agricultural University, Changchun, China; ^3^ Department of Horticulture, Heilongjiang Academy of Agricultural Science, Harbin, China; ^4^ College of Life and Health, Dalian University, Dalian, China

**Keywords:** *Vaccinium* spp., WRKY transcription factors, gene duplication, expression pattern, abiotic stress, subcellular localization

## Abstract

**Introduction:**

The *WRKY* transcription factor (*TF*) family is one of the largest *TF* families in plants and is widely involved in responses to both biotic and abiotic stresses.

**Methods:**

To clarify the function of the *WRKY* family in blueberries, this study identified the *WRKY* genes within the blueberry genome and systematically analyzed gene characteristics, phylogenetic evolution, promoter *cis*-elements, expression patterns, and subcellular localization of the encoded products.

**Results:**

In this study, 57 *VcWRKY* genes were identified, and all encoding products had a complete *WRKY* heptapeptide structure and zinc-finger motif. The *VcWRKY* genes were divided into three subgroups (I-III) by phylogenetic analysis. Group II was divided into five subgroups: IIa, IIb, IIc, IId, and IIe. 57 *VcWRKY* genes were distributed unevenly across 32 chromosomes. The amino acids ranged from 172 to 841, and molecular weights varied from 19.75 to 92.28 kD. Intra-group syntenic analysis identified 12 pairs of duplicate segments. Furthermore, 34 *cis*-element recognition sites were identified in the promoter regions of *VcWRKY* genes, primarily comprising phytohormone-responsive and light-responsive elements. Comparative syntenic maps were generated to investigate the evolutionary relationships of *VcWRKY* genes, revealing the closest homology to dicotyledonous *WRKY* gene families. *VcWRKY* genes were predominantly expressed in the fruit flesh and roots of blueberries. Gene expression analysis showed that the responses of *VcWRKY* genes to stress treatments were more strongly in leaves than in roots. Notably, *VcWRKY13* and *VcWRKY25* exhibited significant upregulation under salt stress, alkali stress, and saline-alkali stress, and *VcWRKY1* and *VcWRKY13* showed notable induction under drought stress. Subcellular localization analysis confirmed that *VcWRKY13* and *VcWRKY25* function within the nucleus.

**Conclusion:**

These findings establish a foundation for further investigation into the functions and regulatory mechanisms of *VcWRKY* genes and provide guidance for selecting stress-tolerant genes in the development of blueberry cultivars.

## 1 Introduction

Blueberries, a small-berry perennial plant in the *Vaccinium* spp. genus in the Ericaceae family, are rich in anthocyanidins, flavonoids, polyphenols, and antioxidants with significant medicinal and health benefits. These bioactive compounds are associated with cardiovascular disease prevention, anti-aging effects, blood sugar reduction, and improve vision (Colle et al., 2019; Duan et al., 2022). Despite their valuable properties, blueberries, as a shrub species with shallow roots, encounter challenges from abiotic stresses like salt, alkali, and drought stress (Chen et al., 2017). For example, salt stress hindered plant growth by inducing osmotic stress and ion toxicity, particularly the excessive accumulation of Na^+^ and Cl^−^ in plant tissues (Van Zelm et al., 2020). Blueberries are acidophilic plants that require acidic soil with pH levels ranging from 4.0 to 5.5, along with adequate drainage and high organic matter content for optimal growth (Zeng et al., 2019). Elevated pH levels in the rhizosphere can diminish nitrogen uptake and impede plant growth due to the rapid conversion of NH_4_^+^-N into NO_3_^–^−N by soil microorganisms (Jiang, 2014; Xu et al., 2021). Furthermore, the shallow roots (less than 40 cm) and lack root hairs restrict blueberries’ capacity to absorb water and nutrients, particularly under drought conditions (Molnar et al., 2022). Notwithstanding these difficulties, not much is known about how tolerance blueberries are to abiotic stress, and it is still unclear what mechanisms underlie stress tolerance. The genetic variability that exists within the blueberry gene pool may be used to enhance its ability to withstand abiotic stresses (Lobos and Hancock, 2015). Therefore, it is critical to exploit stress tolerance-related genes and understand the mechanisms in blueberry plants.

The *WRKY* transcription factor family, one of the largest in plants, plays a crucial role in regulating plant responses to biotic and abiotic stresses (Bakshi and Oelmüller, 2014). WRKY proteins are DNA-binding proteins characterized by a highly conserved domain consisting of about 60 amino acids. They feature a conserved seven-peptide motif, WRKYGQK, at the N-terminus and a zinc-finger structure, either C_2_H_2_ or C_2_HC, at the C-terminus (Jiang et al., 2017). WRKY proteins can be divided into three main subgroups: I, II, and III, based on the number of WRKY domains and the structural characteristics of the zinc-finger motif (Eulgem et al., 2000). Group I members possess two WRKY domains, while members of groups II and III each contain only one WRKY domain. Group II can be subdivided into five subgroups: IIa, IIb, IIc, IId, and IIe (Zhang and Wang, 2005). WRKY proteins specifically bind to the (T)TGAC(C/T) sequence, known as the W-box *cis*-element, in the promoter of target genes through both terminals of the WRKY domain to induce gene expression and maintain cellular homeostasis (Wani et al., 2021). As a result, WRKY proteins can interact with clusters of W-boxes and other elements in the promoters of various genes to regulate a dynamic network of signals and associated physiological responses (Cheng et al., 2021).

*WRKY* genes are known to play crucial roles in various pathways that respond to different abiotic stresses, including cold, drought, UV-B radiation, high temperature, salt, and alkaline conditions (Liu et al., 2021). When plants face adverse environmental conditions, such as osmotic stress, *WRKY* transcription factors modulate downstream gene expression by either repressing or activating target genes through binding to their promoter regions. *WRKY* play a key role in regulating stress-sensory pathways and precise response towards stress (Liu et al., 2014; Wani et al., 2018). In wheat, 47 *WRKY* genes were identified to be expressed under salt stress conditions (Hassan et al., 2019). The *IbWRKY47* gene had been shown to positively regulate stress resistance-related genes and significantly enhance salt tolerance in *Ipomoea batatas* (Qin et al., 2020). In apple (*Malus×domestica*), the regulation of salt tolerance was linked to *MiR156/SPL*, which upregulated the salt tolerance gene *MdWRKY100* (Ma et al., 2021). Overexpression of *MbWRKY4* in transgenic tobacco plants increased salt tolerance and upregulated the expression of oxidative stress response genes (*NtPOD, NtAPX*, and *NtSOD*) under high salinity treatment (Han et al., 2018). A gene encoding a specific transcription factor, along with its target genes, constitutes a regulon that participates in signal transduction to activate or silence genes involved in response to drought (Gahlaut et al., 2016). For instance, the overexpression of *OsWRKY45* in *Arabidopsis* increased drought tolerance by modulating genes related to stomatal closure and stress responses (Qiu and Yu, 2009). Similarly, the overexpression of *TaWRKY146* in *Arabidopsis* enhanced drought tolerance, characterized by elevated levels of proline and soluble sugar, reduced malondialdehyde (MDA) content, stomatal closure, and a decreased transpiration rate (Ma et al., 2017). Liu et al. (2023) furthermore, the co-inhibition of *GmNAC29*, a negative stress response regulator, by *GmWRKY27* and *GmMYB174* improved salt and drought tolerance in transgenic soybean (Wang et al., 2015). Additionally, *PbrWRKY53* in *Pyrus betulaefolia*, *FvWRKY42* in diploid woodland strawberry, and *VvWRKY11* in grape displayed responses to salt, alkali, and drought stresses (Liu et al., 2011; Wei et al., 2018; Liu et al., 2019).

As the number of plants subjected to whole genome sequencing has increased, various members of the *WRKY* transcription factor family have been identified in different species, including *Arabidopsis*(74), maize(119), rice(102), *Oryza rufipogon*(101), safflower(82), *Miscanthus sinensis*(179), chickpea(70), cucumber(55), tomato(81), and watermelon(63) (Ross et al., 2007; Berri et al., 2009; Ling et al., 2011; Huang et al., 2012; Wei et al., 2012; Yang et al., 2018; Waqas et al., 2019; Nan et al., 2020; Song et al., 2023; Yan et al., 2024). However, there remains a notable gap in the comprehensive analysis and functional research of the *WRKY* family in blueberry (*Vaccinium* spp.). To address this, the *WRKY* gene family was identified and analyzed using bioinformatics methods based on the genomic data of blueberry. The structures and promoters of 57 VcWRKY genes were examined, and the conserved domains and motifs of the proteins they encode were identified. Quantitative real-time reverse transcription PCR (RT-qPCR) was used to analyze the expression levels of *VcWRKY* genes under salt, alkali, saline-alkali, and drought treatments, thereby clarifying the regulatory roles of these genes in response to abiotic stress. These findings establish a theoretical foundation for further research on the function of *VcWRKY* genes and provide a new perspective for the development of stress-resistant blueberry cultivars.

## 2 Materials and methods

### 2.1 Genome-wide identification of *WRKY* genes in blueberry

The genome sequence of blueberry (*Vaccinium corymbosum* cv. Draper v1.0) was obtained from the Genome Database for *Vaccinium* (GDV) (https://www.vaccinium.org/crop/blueberry) (Colle et al., 2019). The Hidden Markov model (HMM) file for the WRKY domain (PF03106) was sourced from the PFAM database. The WRKY domain was utilized to identify possible candidate amino acid sequences of *VcWRKY* genes. Each putative *VcWRKY* gene was analyzed using Blastp and NCBI-CDD, which confirmed the presence of the WRKY conserved domain (Lu et al., 2020; Mistry et al., 2021). *VcWRKY* genes were identified by excluding sequences with incomplete or missing domains. ExPASy-ProtParam (https://web.expasy.org/protparam/) was utilized to predict the coding sequence (CDS) length, isoelectric point (pI), molecular weight (MW), and overall average hydrophobicity (GRAVY) of each putative VcWRKY proteins. Additionally, Cell-Ploc (http://www.csbio.sjtu.edu.cn/bioinf/Cell-PLoc-2/) was used to predict the subcellular localization of these putative *VcWRKY* genes.

### 2.2 Phylogenetic analysis, gene structure, conserved motifs, and *cis*-acting elements

The WRKY protein sequences of *Arabidopsis thaliana*, *Actinidia chinensis, Malus domestica*, and *Solanum lycopersicum* were obtained from the PlantTFDB database. Using the neighbor-joining method with MEGA 7.0 software, a phylogenetic tree of *WRKY* gene families in blueberry and these four other species was constructed with 1000 bootstrap replications. For the multiple sequence alignments of *VcWRKY* genes, a 60-amino acid sequence containing WRKY domains and zinc-finger motifs was extracted. The gene structure and conserved domains of *VcWRKY* genes were analyzed and visualized using Web CD-search and TBtools (Chen et al., 2020). Conserved motifs of putative *VcWRKY* genes were identified using MEME (http://meme.nbcr.net/meme/tools/meme), with a maximum search value for conserved motif set to 10. TBtools was utilized to integrate the evolutionary tree of *VcWRKY* genes with conserved domains and motifs. The 2000-bp sequence upstream of each *VcWRKY* genes coding sequence (CDS) was extracted as the promoter region, and promoter *cis*-elements were predicted using PlantCare (http://bioinformatics.psb.ugent.be/webtools/plantcare/html/). The results were visualized with TBtools after statistical screening.

### 2.3 Chromosomal distribution of *VcWRKY* genes

*VcWRKY* genes were mapped to the chromosome based on the blueberry genomic data. The analysis of tandem and segmental duplication events of the *VcWRKY* genes was conducted using the Multiple Collinearity Scan toolkit X (MCScanX) with default parameters. Synteny analysis was carried out in blueberry and seven other species (*Arabidopsis thaliana, Solanum lycopersicum, Actinidia chinensis, Vitis vinifera, Oryza sativa, Prunus persica*, and *Zea mays*), with TBtools used for visualization. Additionally, TBtools was used to calculate the non-synonymous (Ka) and synonymous (Ks) ratio for duplicated gene pairs.

### 2.4 MiRNA prediction and SSR evaluation of *VcWRKY* genes

To predict miRNAs that may target *VcWRKYs* in blueberry, the sequences of 638 miRNAs reported in evergreen blueberry (*Vaccinium darrowii*) (Yu et al., 2021) and 57 *VcWRKY* genes identified in this study were input into psRNATarget (Dai and Zhao, 2011). Additionally, Krait software (Du et al., 2017) was utilized to analyze the 57 *VcWRKY* sequences to explore the distribution of SSR loci within *VcWRKY*s.

### 2.5 Plant materials and stress treatments

Different tissues of the blueberry cultivar ‘Northland’-including leaf buds, flower buds, leaves, flowers, fruit, seeds, roots, and stems-were collected at the Small Berry Germplasm Resources Nursery of Jilin Agricultural University. Three-year-old blueberry seedlings, grown from cuttings and belonging to the cultivar ‘Northland’, were selected for abiotic stress treatment. All selected seedlings exhibited similar size and plant height. The seedlings were obtained from Heyun Modern Agriculture Co., Ltd., Tonghua, Jilin, China. Before stress treatments, the plants (3-year-old blueberry clones) were acclimatized in hydroponic incubators for 15 days. The nutrient solution in the 4 L hydroponic incubators was continuously aerated and contained 10 mmol/L NH_4_NO_3_, 5 mmol/L K_2_SO_4_, 1.25 mmol/L KH_2_PO_4_, 1.5 mmol/L MgSO_4_·7H_2_O, 100 μmol/L H_3_BO_3_, 75 μmol/L MnSO_4_·4H_2_O, 30 μmol/L ZnSO_4_·7H_2_O, 1 μmol/L Na_2_MO_4_·2H_2_O, 0.1 μmol/L CuSO_4_·5H_2_O, 0.1 μmol/L FeSO_4_·7H_2_O, 0.1 μmol/L Na_2_EDTA·2H_2_O, and 0.47 μmmol/L Ca(NO_3_)_2_·4H_2_O. During this acclimatization period, the nutrient solution was changed every 3 days to facilitate the plants’ adaptation to the hydroponic environment.

All plants were kept in a greenhouse with a temperature of 25 ± 1°C and 50% relative humidity, with a photoperiod of 16 hours of light followed by 8 hours of darkness. The abiotic stress treatments were designed using a fully randomized trial approach, which included salt stress (110 mM NaCl), alkali stress (110 mM NaHCO_3_), saline-alkali stress (50 mM NaCl + 70 mM NaHCO_3_), and drought stress simulated by polyethylene glycol (8% PEG8000). For each stress treatment, 1 L of the treatment solution was added to each incubator to reach the specified concentration levels. Leaf and root samples were collected at 0, 3, 6, 9, and 12 hours following the initiation of each stress treatment. Three biological replicates were taken for each sampling time point, and all samples were immediately frozen in liquid nitrogen and stored at -80°C until analysis.

### 2.6 Total RNA extraction and quantitative real-time PCR analysis

Blueberry samples (0.1 g) were ground in liquid nitrogen, and total RNA was extracted using the E.Z.N.A.^®^ Plant RNA Kit (Omega). The integrity, concentration, and quality of the RNA were assessed through 1% agarose gel electrophoresis and spectrophotometry (Implen Ultramicro). cDNA was synthesized using the PrimeScript™ RT reagent kit and gDNA Eraser (For perfect real time) kit (TaKaRa), with a ten-fold dilution of cDNA used for RT-qPCR and stored at -20°C. RT-qPCR primers were designed using Primer 3.0, based on the gene structure of *VcWRKYs* (**Supplementary Table S1**). Referring to our previous study (Deng et al., 2020), *elongation factor 1-alpha 3 (EF1α)* was used as the reference gene for RT-qPCR analyses. The protocol of the TB Green™ Premix Ex Taq™II kit (Tli RNaseH Plus) was adhered to for RT-qPCR. The RT-qPCR reaction system included template cDNA (20 ng/μL) 1 µL, each primer (10 µM) 0.4 µL, ddH_2_O 3 µL, TB Green Premix Ex Taq II (Tli RNaseH Plus) (*2×*) 5 μL, and ROX Reference Dye (*50×*) 0.2 μL. The reaction program was as follows: pre-denaturation at 95°C for 30 s; denaturation at 95°C for 5 s, annealing at 60°C for 30 s, and extension at 72°C for 10 s, for a total of 40 cycles. RT-qPCR was conducted using the qTOWER3G touch real-time system (Analytik Jena, Germany), with three biological replicates and three technical repeats for each sample. Relative expression levels of *VcWRKYs* under different stress treatments were calculated using the 2^-ΔΔCt^ method and visualized using Origin and TBtools.

### 2.7 Plasmid construction and subcellular localization analysis

The highly expressed *VcWRKY13* and *VcWRKY25* genes, which respond to four different abiotic stresses, were selected for gene cloning and subcellular localization. Using Primer 3.0, cloning primers were designed based on the coding sequences (CDS) of *VcWRKY13* and *VcWRKY25* (**Supplementary Table S2**). Subsequently, the CDS of VcWRKY13 and VcWRKY25 were amplified from cDNA derived from RNA extracted from blueberry leaves. The PCR mixture (50 µL) included Ex Taq^®^DNA polymerase (5 U/μL) 0.25 μL (TaKaRa, Dalian, China), *10×*Ex Taq Buffer (Mg^2+^ plus) (20 mM) 5 μL, dNTP mixture (2.5 mM) 4 μL, each primer (10 µM) 2 µL, cDNA 2 μL, and ddH_2_O up to 50 μL. The PCR program included pre-denaturation at 94°C for 5 min; denaturation at 94°C for 30 s, annealing at 55°C for 30 s, extension at 72°C for 1:10 min, repeated for 35 cycles; followed by a final extension at 72°C for 7 minutes. The PCR products were purified using a DNA Gel Extraction Kit (AxyPrep, Union City, CA, USA), ligated into the pMD™18-T vector (TaKaRa), and sequenced.

The coding sequences (CDS) of *VcWRKY13* and *VcWRKY25*, excluding the stop codon, were amplified and inserted into the transient expression pGDG vector, which contained a GFP fluorescent label and a CaMV35S promoter. The primer sequences used for plasmid construction were detailed in **Supplementary Table S3**. Each correctly constructed plasmid was then inserted into *Agrobacterium* GV3101 using the conventional freezing-thawing method. Empty pGDG-GFP or pGDG-VcWRKYs-GFP GV3101 were infiltrated into the leaves of 5-week-old *N. benthamiana* plants with an expression buffer (10 mM MES pH 5.6, 10 mM MgCl_2_, 150 µM acetosyringone). Following infiltration, the *N. benthamiana* plants were kept in darkness for 24 hours and then under low light for 48 hours. GFP signals were observed using a fluorescence microscope (Echo Revolve, Echo Laboratories, San Diego, CA, USA). The DAPI staining was used as a nucleus marker for nucleus detection.

### 2.8 Statistical analysis

The relative expression of the target gene was determined using the 2^−ΔΔCT^ method. Statistical analysis was carried out using SPSS version 24.0, with Duncan’s test utilized to evaluate the significant differences among all samples (^*^p ≤ 0.05, ^**^p ≤ 0.01, ^***^p ≤ 0.001). Data visualization was accomplished using Origin 2021. To ensure reliability, three biological replicates were conducted for each sample.

## 3 Results

### 3.1 Identification of *VcWRKY* genes in blueberry

Using the blueberry genome and the WRKY domain model (PF03106), 57 *VcWRKY* genes were identified and designated as *VcWRKY1*-*VcWRKY57*. These 57 VcWRKY proteins were analyzed for molecular weight (MW), theoretical isoelectric point (pI), and subcellular localization (**Supplementary Tables S4**, **S5**). The lengths of the VcWRKY proteins varied from 172 amino acids (VcWRKY36) to 841 amino acids (VcWRKY13). The molecular weights ranged from 19.75 kD (VcWRKY36) to 92.28 kD (VcWRKY13), and the theoretical isoelectric points (pI) varied from 4.42 (VcWRKY43) to 9.77 (VcWRKY30). The mean hydrophilicity values of the VcWRKY proteins were negative, indicating their hydrophilic nature. Additionally, subcellular localization predictions revealed that all VcWRKY proteins were localized in the nucleus.

### 3.2 Phylogenetic analysis and multiple sequence alignment of VcWRKY proteins

**Figure 1:**
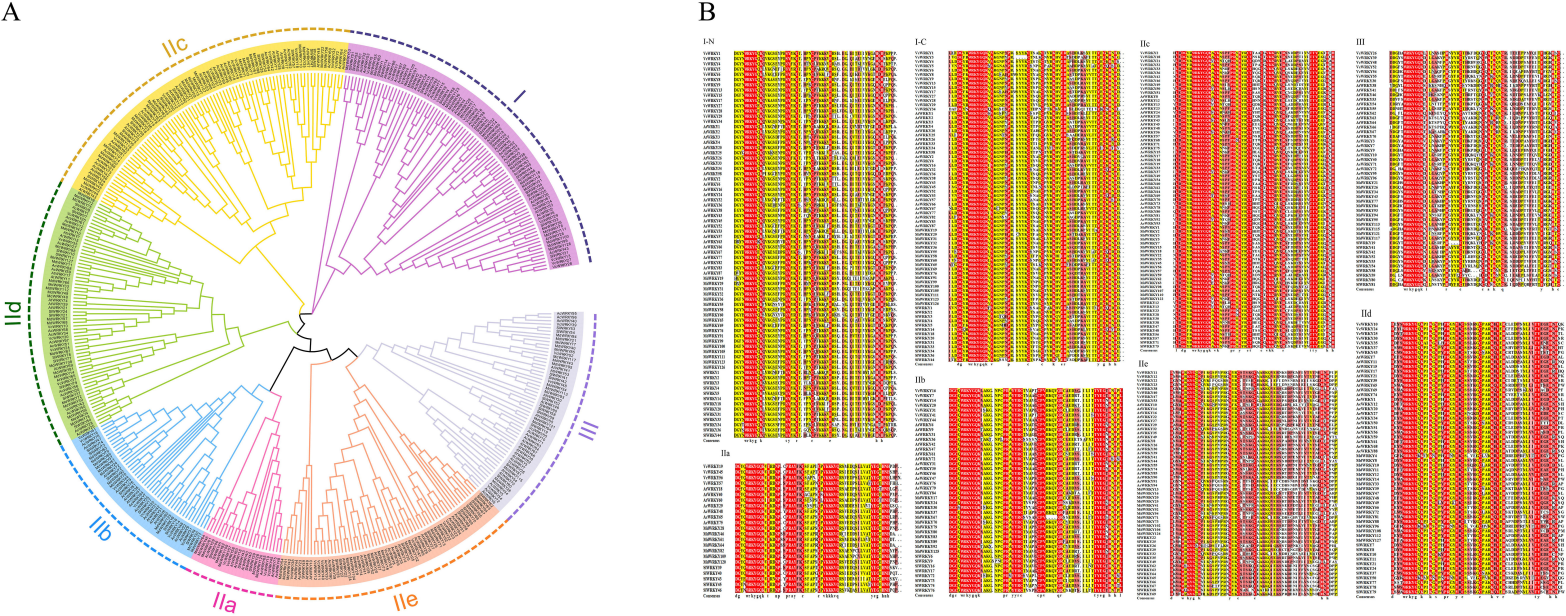
Phylogenetic analysis and multiple sequence alignment of *VcWRKYs*. **(A)** Phylogenetic tree of the relationships between the WRKY proteins of *Vaccinium*, *Arabidopsis thaliana*, *Actinidia chinensis, Malus domestica*, and *Solanum lycopersicum*. **(B)** Multiple sequence alignment of the WRKY domains of the blueberry and four other species.

To examine the evolutionary relationship among the *WRKY* gene family in *Vaccinium* (57 *VcWRKYs*), *Arabidopsis thaliana* (66 *AtWRKYs*), *Actinidia chinensis* (80 *AcWRKYs*)*, Malus domestica* (100 *MdWRKYs*), and *Solanum lycopersicum* (74 *SlWRKYs*), a phylogenetic tree was constructed using the NJ technique in MEGA7.0 (**Figure 1A**). 57 VcWRKY proteins were divided into three subgroups (I, II, and III), representing 24.56%, 64.91%, and 10.53%, respectively. Group II was subdivided into five subgroups: IIa, IIb, IIc, IId, and IIe, containing 4, 7, 11, 7 and 8 members, respectively. To further explore the evolutionary relationship of the structural domains of the VcWRKY proteins within the subgroups, multiple sequence comparisons were conducted on the amino acid sequences of the VcWRKY proteins alongside those from four other species, focusing on 60 amino acid sequences surrounding the WRKY domains (**Figure 1B**). The analysis revealed a high level of conservation in the WRKY heptapeptide structure at the N-terminus and the zinc-finger motif at the C-terminus. The VcWRKY proteins in group I exhibited two WRKY domains and the CX_4_CX_23_HXH-type zinc-finger motif. In contrast, all other VcWRKY proteins in group II displayed the CX_5_CX_23_HXH-type zinc-finger motif, except for group IIc, which had the CX_4_CX_23_HXH-type. Group III VcWRKY proteins showed the CX_7_CX_23_HXC-type zinc-finger motif.

### 3.3 Gene structures, conserved motifs and predicted 3D model of *VcWRKY* genes

**Figure 2:**
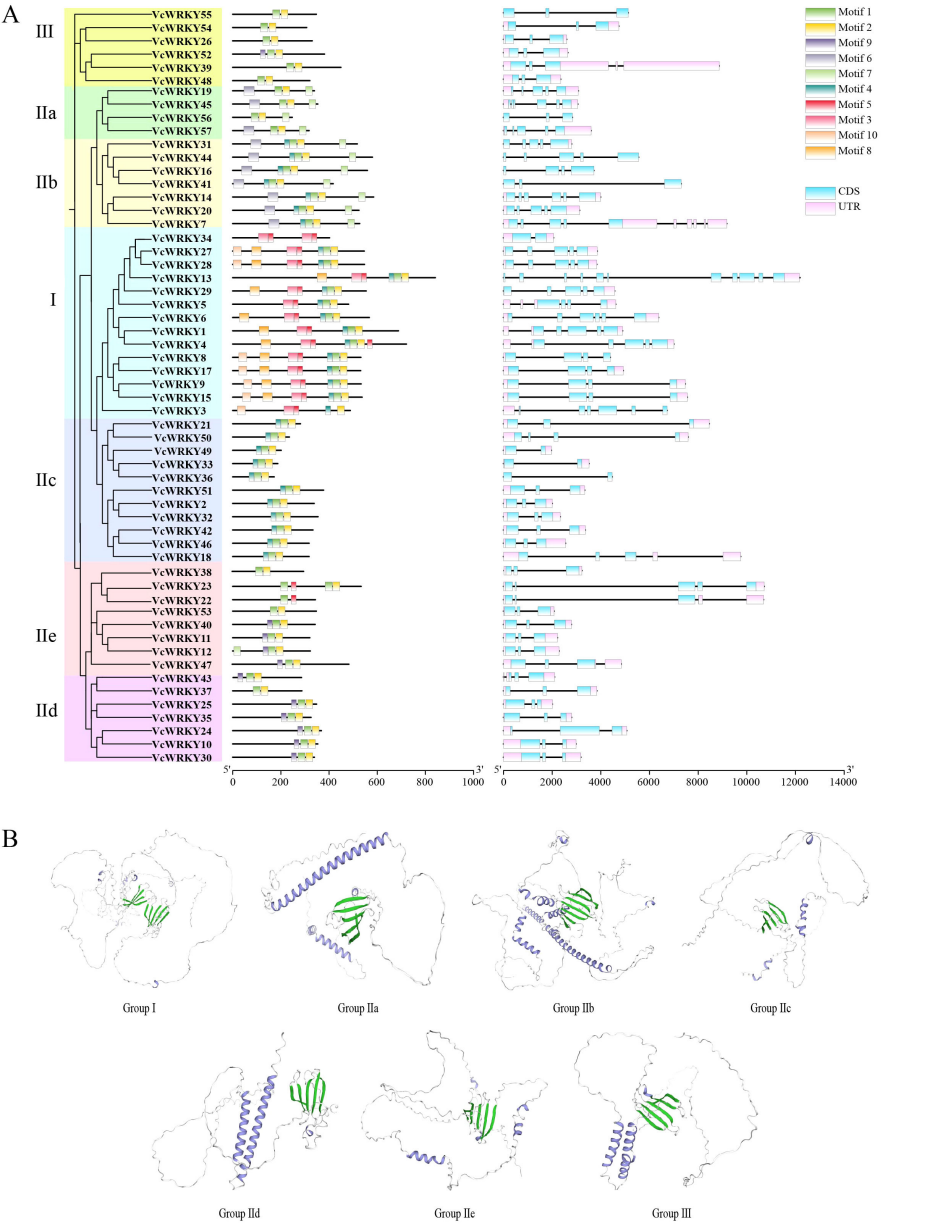
Structural analysis of VcWRKYs. **(A)** Phylogenetic tree, motif pattern, and gene structure of VcWRKY proteins. **(B)** Predicted 3D structure model of VcWRKYs representing different subgroups were analyzed by Swiss Model.

Using the blueberry genome sequence, a structural map was made to illustrate the coding sequence (CDS) and untranslated region (UTR) of each *VcWRKY* gene (**Figure 2A**). The *VcWRKY* genes exhibited a range of two to eleven exons and two to seven introns, with similar exon/intron distribution patterns observed within the same subgroups. UTRs were identified in 51 *VcWRKY*s, with the exception of *VcWRKY16/36/41/44/55/56*. All 57 *VcWRKY* genes contained exons: 29 genes had three exons, 11 genes had five exons, 8 genes had four exons, and only *VcWRKY13* had eleven exons. MEME software identified 10 conserved motifs among the 57 VcWRKY proteins, with motif 1 representing the WRKY domain and motif 2 corresponding to the zinc-finger motif (**Figure 2A**). Both motifs 1 and 2 were present in all VcWRKY proteins. Motifs 6 and 7 were predominantly found in groups IIa and IIb, while motifs 3, 5, 8, and 10 were prevalent in groups I and IIe. Motif 4 was distributed across groups I, IIb, and IIc, whereas both groups I and IId contained motif 9. Additionally, the 3D structural models of VcWRKYs, representing different subgroups, were predicted using Swiss Model (**Figure 2B**). VcWRKYs of Group I contained two WRKY domains, with one domain characterized by four beta-sheets and the other by five beta-sheets. In contrast, the remaining groups possessed only a single WRKY domain. Members of groups IIa, IIb, and IIc exhibited an alpha coil at the C-terminus (a transcriptional activation domain). The group IIe model displayed a short alpha coil located between the first and last beta-sheets.

### 3.4 Promoter *cis*-elements analysis of *VcWRKY* genes

**Figure 3:**
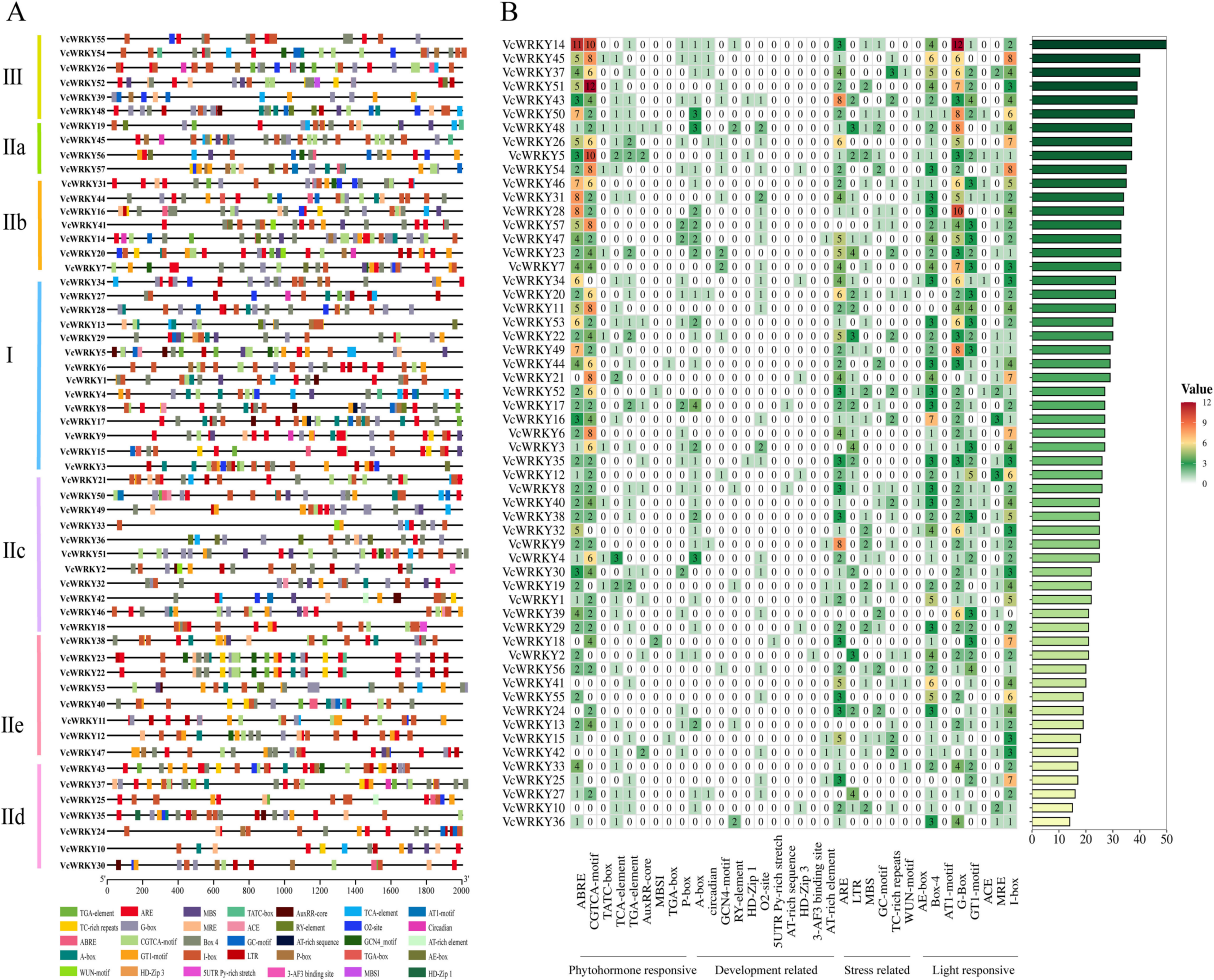
The *cis*-acting element of the promoter region (upstream 2000 bp) of *VcWRKY* genes. **(A)** Various types of *cis*-elements and their respective locations in each *VcWRKY* genes. **(B)** The numbers of different *cis*-acting elements in the initiation regions of *VcWRKY* genes.

The WRKY proteins specifically recognize and interact with various *cis*-elements within the promoter regions of target genes to carry out diverse biological functions. A comprehensive analysis of the *cis*-elements present in the promoter region of *VcWRKY* genes was performed to gain insights into their biological roles and underlying molecular mechanisms. Among the 57 *VcWRKY* genes analyzed, we identified 34 different *cis*-elements located 2000 bp upstream of the start codon (ATG) (**Figure 3**; **Supplementary Table S6**). These *cis*-elements were associated with phytohomone responsive, development related, stress related, and light responsive. The phytohormone responsive elements included important motifs such as abscisic acid responsiveness (ABRE), gibberellin responsive elements (P-box, TATC-box), MeJA responsiveness (CGTCA-motif, TGACG-motif), auxin responsive element (TGA-element) and salicylic acid responsiveness (TCA-element). Notably, MeJA and ABA responsiveness were the most prevalent among the identified *cis*-elements. Particularly, *VcWRKY5/14/26/45/51* exhibited a high abundance of phytohormone responsive elements, suggesting their potential pivotal roles in phytohormone signaling pathways. Conversely, *cis*-elements associated with plant growth and development were relatively scarce, including metabolism regulation responsive (O2-site), endosperm expression responsive (GCN4_motif), seed-specific regulation responsive (RY-element), circadian rhythm control responsive (circadian). Stress-responsive *cis*-elements included motifs such as anaerobic induction responsiveness (ARE), drought inducibility responsiveness (MBS), low temperature responsiveness (LTR), and defense and stress responsiveness (TC-rich repeats). Notably, a significant number of *cis*-acting elements related to light responsiveness were identified, including I-box, G-box, Box-4, and GT1-motif. Furthermore, we predicted the presence of W-box recognition sites, which served primary targets for *WRKY* transcription factors.

### 3.5 Chromosome distribution, gene duplication of *VcWRKY* genes

**Figure 4:**
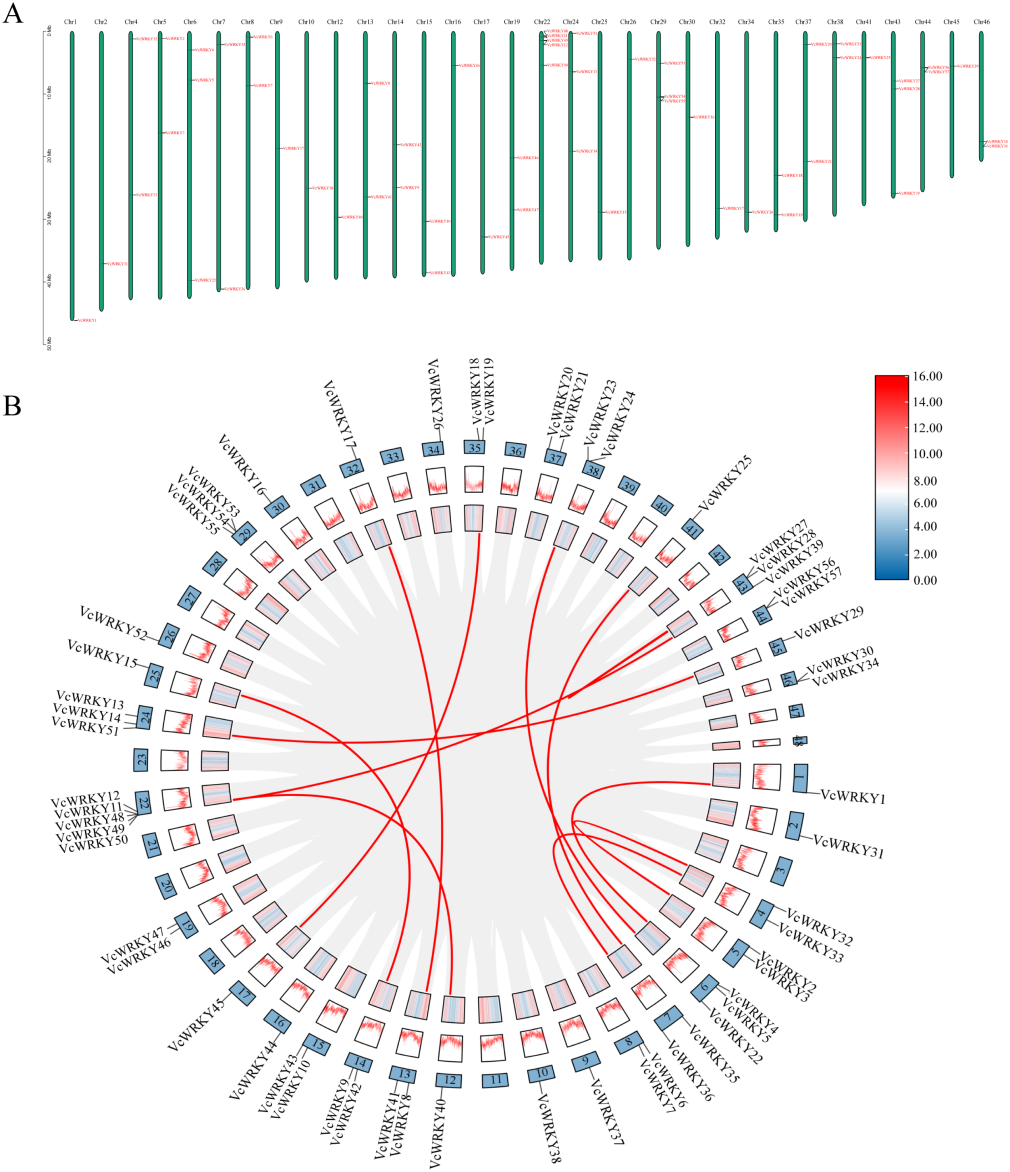
The distribution information of *VcWRKY* genes on chromosomes and gene replication events in the blueberry. **(A)** Chromosomal location of *VcWRKY* genes. **(B)** Chromosome distribution and gene duplication relationship of *VcWRKY* genes. The gray lines represent the synteny regions in the blueberry genome. The red lines represent syntenic *VcWRKY* gene pairs.

A total of 57 *VcWRKY* genes were distributed unevenly across 32 chromosomes; specifically, Chr22 had five genes, while Chr6, Chr24, Chr29, and Chr43 each had three genes (**Figure 4A**). Thirteen chromosomes had two genes, and the remaining fourteen chromosomes each had one gene. Group I WRKY genes were located on Chr1, Chr6, Chr25, Chr32, and Chr45, whereas group II WRKY genes were found on Chr2, Chr4, Chr7, Chr9, Chr12, Chr15, Chr16, Chr17, Chr19, Chr30, Chr35, Chr37, Chr41, and Chr44. Group III WRKY genes were present on Chr10, Chr26, and Chr34. Additionally, the distribution of WRKY genes from different groups was mixed across several chromosomes: groups I and II were located on Chr5, Chr8, Chr13, Chr14, Chr24, Chr38, and Chr46; group II and III were found on Chr22 and Chr29; groups I and III were identified on Chr13.

During the segmental duplication analysis of the 57 *VcWRKY* genes, twelve pairs of duplicated genes were identified using BLASTP and MCScanX (**Figure 4B**): *VcWRKY1/VcWRKY4, VcWRKY40/VcWRKY12, VcWRKY8/VcWRKY17, VcWRKY9/VcWRKY15, VcWRKY45/VcWRKY19, VcWRKY48/VcWRKY39, VcWRKY13/VcWRKY29, VcWRKY23/VcWRKY22, VcWRKY32/VcWRKY2, VcWRKY33/VcWRKY36, VcWRKY25/VcWRKY35, VcWRKY27/VcWRKY28*. Each gene involved in tandem duplication events belongs to the same subgroup. Group I consisted of six pairs of *VcWRKY* genes, while groups IIa, IId, IIe, and III each included one pair of duplicates. Group IIc comprised two pairs of duplicates. To verify whether these gene pairs underwent purifying selection, we calculated the non-synonymous substitution rate (Ka) and synonymous substitution rate (Ks). All homozygous *VcWRKY* gene pairs had Ka/Ks ratios below 1, indicating that these gene pairs had underwent purifying selection (**Supplementary Table S7**).

### 3.6 Synteny analysis of VcWRKY proteins among different plants

**Figure 5:**
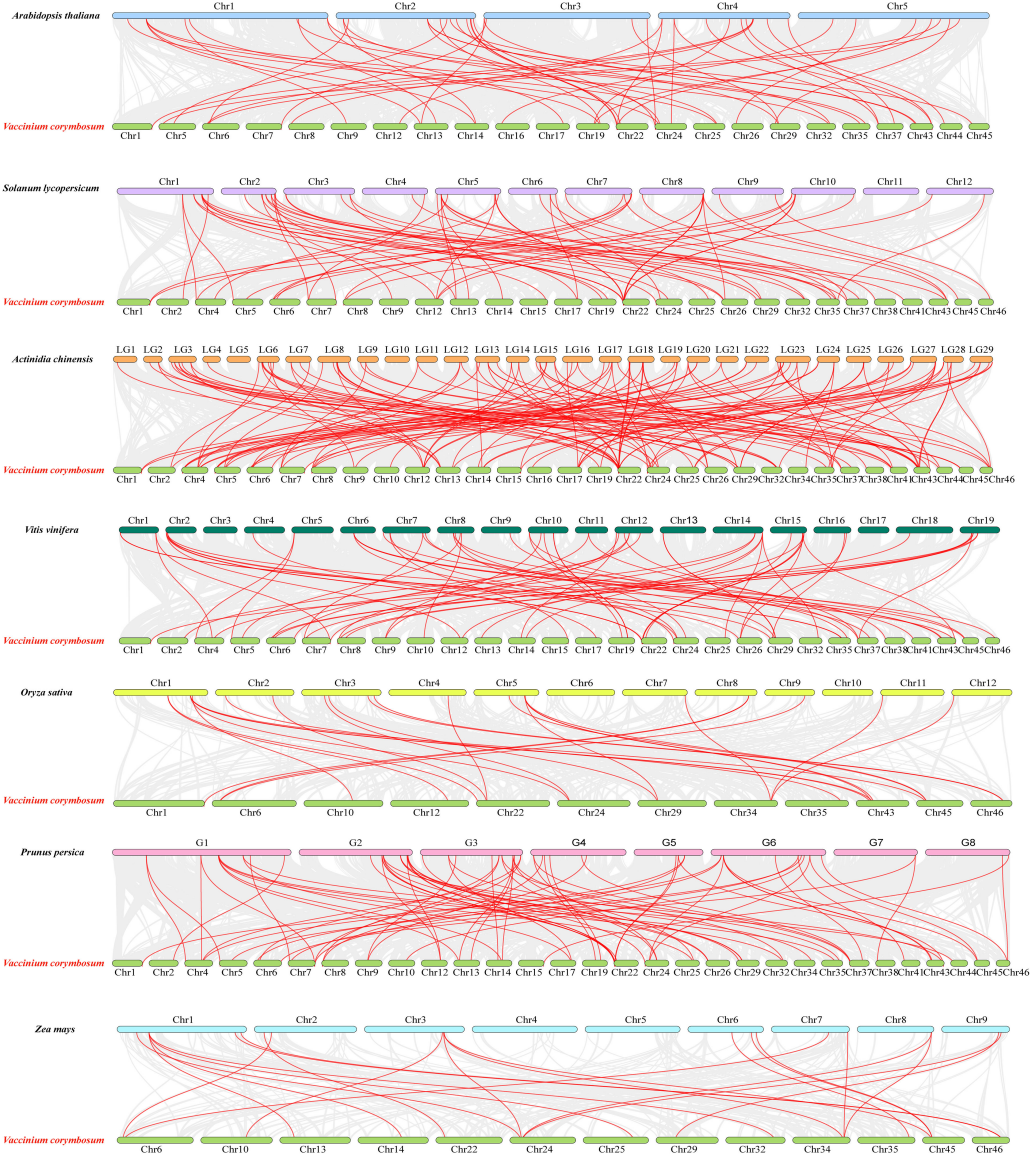
Synteny analysis of *WRKY*s in blueberry and seven other species (*Arabidopsis thaliana, Solanum lycopersicum, Actinidia chinensis, Vitis vinifera, Oryza sativa, Prunus persica*, and *Zea mays*). The grey lines in the background represent synteny blocks in blueberry and seven other species, respectively. The red lines represent syntenic *WRKY* gene pairs. The numbers on the chromosomes indicate chromosome numbers.

To investigate the evolutionary relationship between *VcWRKYs* and different species, synteny maps were constructed for blueberry alongside two monocotyledons (*Oryza sativa* and *Zea mays*) and five dicotyledons (*Arabidopsis thaliana, Vitis vinifera, Actinidia chinensis, Prunus persica*, and *Solanum lycopersicum*) (**Figure 5**). The *VcWRKYs* exhibited a strong synteny relationship with *WRKY* genes of dicotyledonous plants, with the highest number of pairs observed in *Actinidia chinensis* (131 pairs), followed by *Prunus persica* (74 pairs), *Vitis vinifera* (67 pairs), *Solanum lycopersicum* (66 pairs), and *Arabidopsis thaliana* (52 pairs). Certain genes were also shared with the monocotyledons, such as *Zea mays* (22 pairs) and *Oryza sativa* (25 pairs). It was indicated that *VcWRKY* genes showed higher homology with *Actinidia chinensis*, possibly due to their close genetic relationship. Despite chromosomal rearrangements or gene duplications, the synteny analysis of *VcWRKY* genes demonstrated robust synteny.

### 3.7 Identification of miRNAs and SSRs in *VcWRKY* genes

The 20-22 nucleotide microRNAs (miRNAs) play critical roles in both plant development and biotic and abiotic stress responses. MiRNAs regulate gene expression at posttranscriptional levels through specific base-pairing with target mRNAs (Gao et al., 2022). In order to identify the miRNA targets in blueberry, the 57 identified *VCWRKY* genes were analyzed using psRNATarget. A total of 87 unique potential miRNAs targeting 43 *VcWRKY* members were identified (**Supplementary Table S8**). Among these, *VcWRKY27* and *VcWRKY55* were the most frequently targeted genes, each being targeted by seven miRNAs in the predictive analysis. Most of the 57 VcWRKY genes were targeted by miRNAs associated with abiotic stress. For instance, miR2105 (Gao et al., 2022), which was associated with drought stress, targeted *VcWRKY27* and *VcWRKY28* of Group I. *VcWRKY10/30/52* had a target that binds to miR854, which was also related to drought stress (Zhou et al., 2010). Meanwhile, *VcWRKY9/13/15/21/23/27/28/29/38/53* had miR530 binding targets associated with disease resistance and yield (Li et al., 2021). *VcWRKY27/31/39* had targets that bind to miR166, which was associated with drought and salt stress (Li et al., 2022; Zhao et al., 2024). Lastly, miR5207 (Yan et al., 2020) related to salicylic acid synthesis, targeted *VcWRKY6/31/44/45*. Therefore, given that most miRNA targets are conserved in plants, it can be inferred that the expression of blueberry *VcWRKY* genes is regulated by miRNAs.

Marker-assisted selection (MAS) involving SSR markers associated with transcription factor (TF) genes can also be used for the development of abiotic stress resistance genotypes (Gahlaut et al., 2016). To evaluate potential SSR markers in 57 *VCWRKY* genes, predictions were conducted using Krait software. 120 SSR loci were predicted across the 57 sequences, indicating a 2.05% occurrence rate, a relative abundance of 1070.45, and a relative density of 20838.17 (**Supplementary Table S9**). Mononucleotide repeats emerged as the predominant form, constituting 60.0%, followed by dinucleotide repeats at 30.83% and trinucleotide repeats at 4.17%. Tetranucleotide and pentanucleotide SSRs were relatively scarce, accounting for 3.33% and 1.67%, respectively. Among the 22 distinct repeat motifs, AG/CT motifs were the most common at 25.86%, closely followed by A/A motifs at 22.21%. The average length of SSR motifs was 19.47 bp, and SSRs ranging from 7 to 14 bp were the most common, accounting for 56.67%, while those with 18-24 bp were the least, accounting for 3.32%. Among the 57 *VcWRKYs*, except *VcWRKY14/24/43/55*, all other genes contain SSR loci. *VcWRKY42* had the most abundant SSR loci. Eight *VcWRKY* sequences were identified to contain four or more SSR loci, while 17 sequences contained a single SSR loci.

### 3.8 Expression patterns of *VcWRKY* genes in different tissues

**Figure 6:**
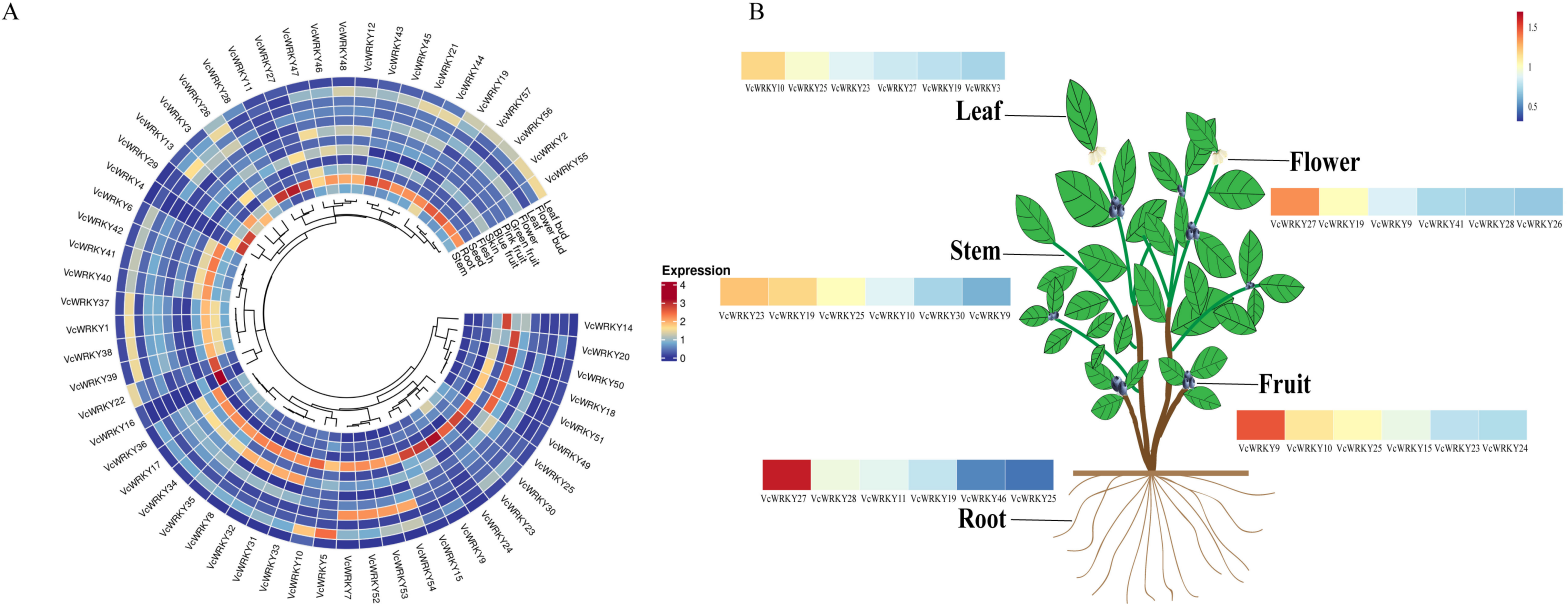
Expression atlas of *VcWRKY* genes in multiple blueberry tissue types. **(A)** Expression patterns of 57 *VcWRKY* genes in 12 tissues. Blue and red colors indicate low-and high-expressed genes, respectively. **(B)** Heatmap of *VcWRKY* genes with high expression levels in blueberry leaves, stems, roots, flowers, and fruits.

To explore the expression patterns of 57 *VcWRKY* genes in blueberries, the expression levels of these genes were analyzed by RT-qPCR in different tissues, including leaf buds, flower buds, leaves, flowers, green fruits, pink fruits, blue fruits, fruit skins, fruit fleshes, seeds, roots, and stems (**Figure 6**). Among the 12 tissues examined, the highest expression levels of *VcWRKY* genes were observed in the fruit fleshes and roots. Genes belonging to the same group had similar expression patterns. *VcWRKY9/10/15/25* showed high expression levels in blue fruits, fruit skins, and fruit fleshes, particularly *VcWRKY9* showed the highest expression in both blue fruits and fruit fleshes. Specifically, the expression level of *VcWRKY9* in fruit fleshes was 94.5-fold higher than in leaf buds, suggesting a potential role in fruit expansion and flavor formation. *VcWRKY10/24/25/30* of group IId exhibited high expression levels in fruit fleshes. Among flower buds, leaves, green fruits, pink fruits, and seeds, *VcWRKY10* displayed the highest expression levels. *VcWRKY25* also showed the highest expression in pink fruits, with similarly elevated levels in other tissues. Furthermore, *VcWRKY27/28* showed high expression levels in roots, and *VcWRKY27* also showed high levels in fruit skins and leaves. This suggested that *VcWRKY27* might be involved in various physiological and biochemical reactions in these tissues. *VcWRKY11* of group IIe showed high expression levels in flower buds and roots. Overall, the majority of *VcWRKY* genes showed varying degrees of expression across multiple organs, with the exception of *VcWRKY16*. Notably, *VcWRKY9/10/27* exhibited higher expression levels than other *VcWRKY* genes in 12 tissues.

### 3.9 Expression patterns of *VcWRKY* genes in leaves under different stress treatments

**Figure 7:**
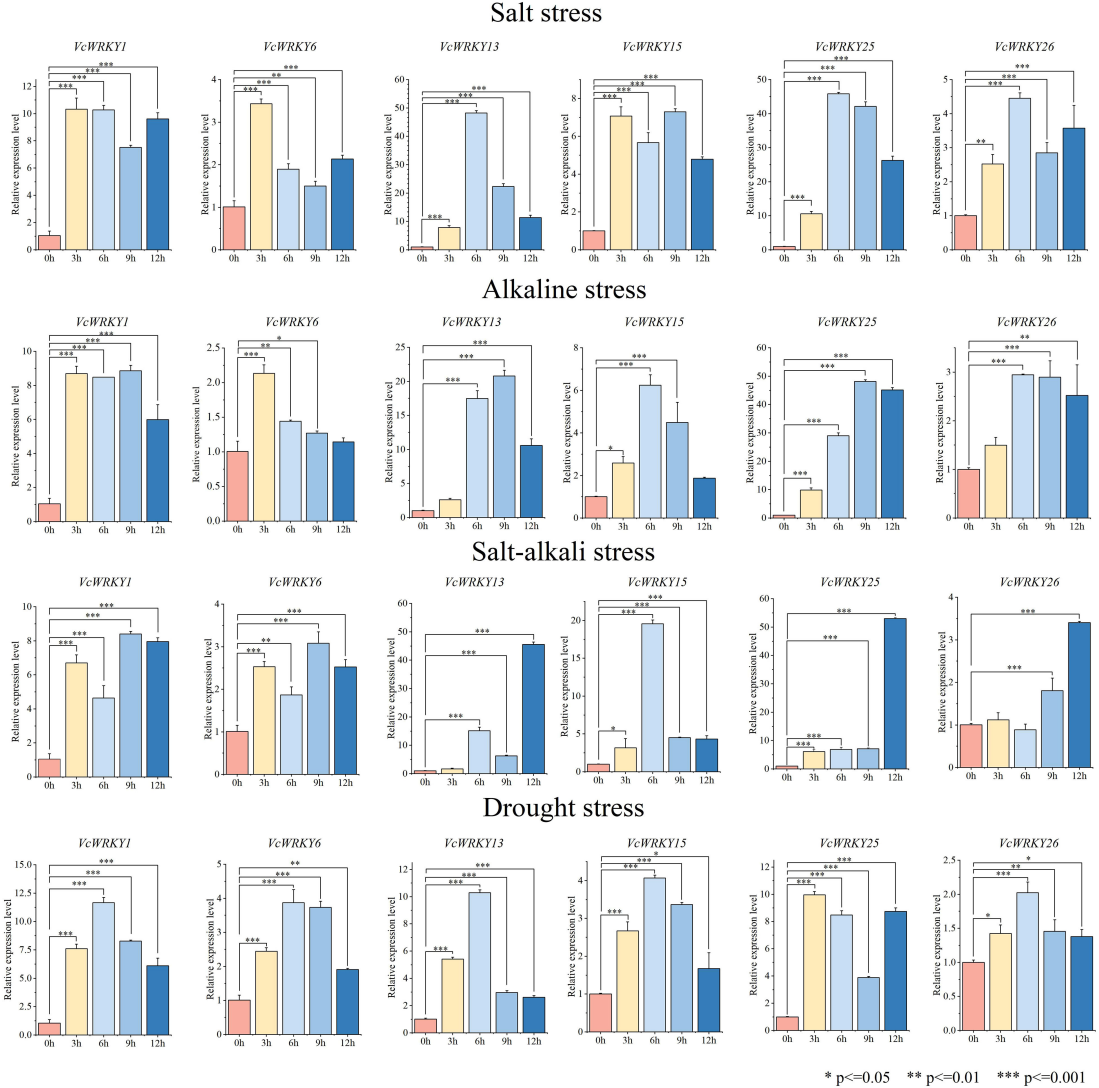
Expression patterns of *VcWRKY* genes in blueberry leaves after four stress treatments. The ^*^ indicates significant differences by t-test (*p≤0.05, **p≤0.01, ***p≤0.001).

To investigate the role of *VcWRKY* genes in the response mechanism to salt stress, alkali stress, salt-alkali stress, and drought stress, blueberry seedlings were treated with each stress. Samples were collected at 0, 3, 6, 9, and 12 h, with the 0-h samples serving as the control. RT-qPCR was used to analyze the expression patterns of six *VcWRKY* genes under different abiotic stresses (**Figure 7**). These *VcWRKY* genes, which belong to different subgroups, had high expression differences under stress treatments and had close evolutionary relationships with stress-related *AtWRKY* genes in *Arabidopsis*. The six *VcWRKY* genes responded differently to salt stress. *VcWRKY1/6* peaked at 3 h, with increased of 10-fold and 3-fold, respectively. *VcWRKY15* had its maximum expression at 9 h, while *VcWRKY13/25/26* peaked at 6 h. Notably, in response to salt stress, *VcWRKY13/25* showed significant upregulation of 48-fold and 45-fold, respectively. Under alkaline stress, *VcWRKY1/13/25* peaked at 9 h, with increased of 8, 20, and 48-fold, respectively. Additionally, *VcWRKY15/26* peaked after 6 h, while *VcWRKY6* peaked after 3 h. In response to salt-alkali stress, *VcWRKY1/6* peaked after 9 h. *VcWRKY13/25/26* peaked at 12 h, with upregulation of 45, 53, and 3-fold, respectively. *VcWRKY15* also showed a strong response to salt-alkali stress, with a 20-fold increase at 6 h. Compared to the other treatments, the *VcWRKY* genes showed weaker responses to the PEG-simulated drought stress. *VcWRKY25* had the lowest expression level during drought stress, just one-fifth of that under salt stress. Interestingly, *VcWRKY1/13* showed a strong response to drought stress, with increased of 11 and 10-fold, respectively.

### 3.10 Expression patterns of *VcWRKY* gene in roots under different stress treatments

**Figure 8:**
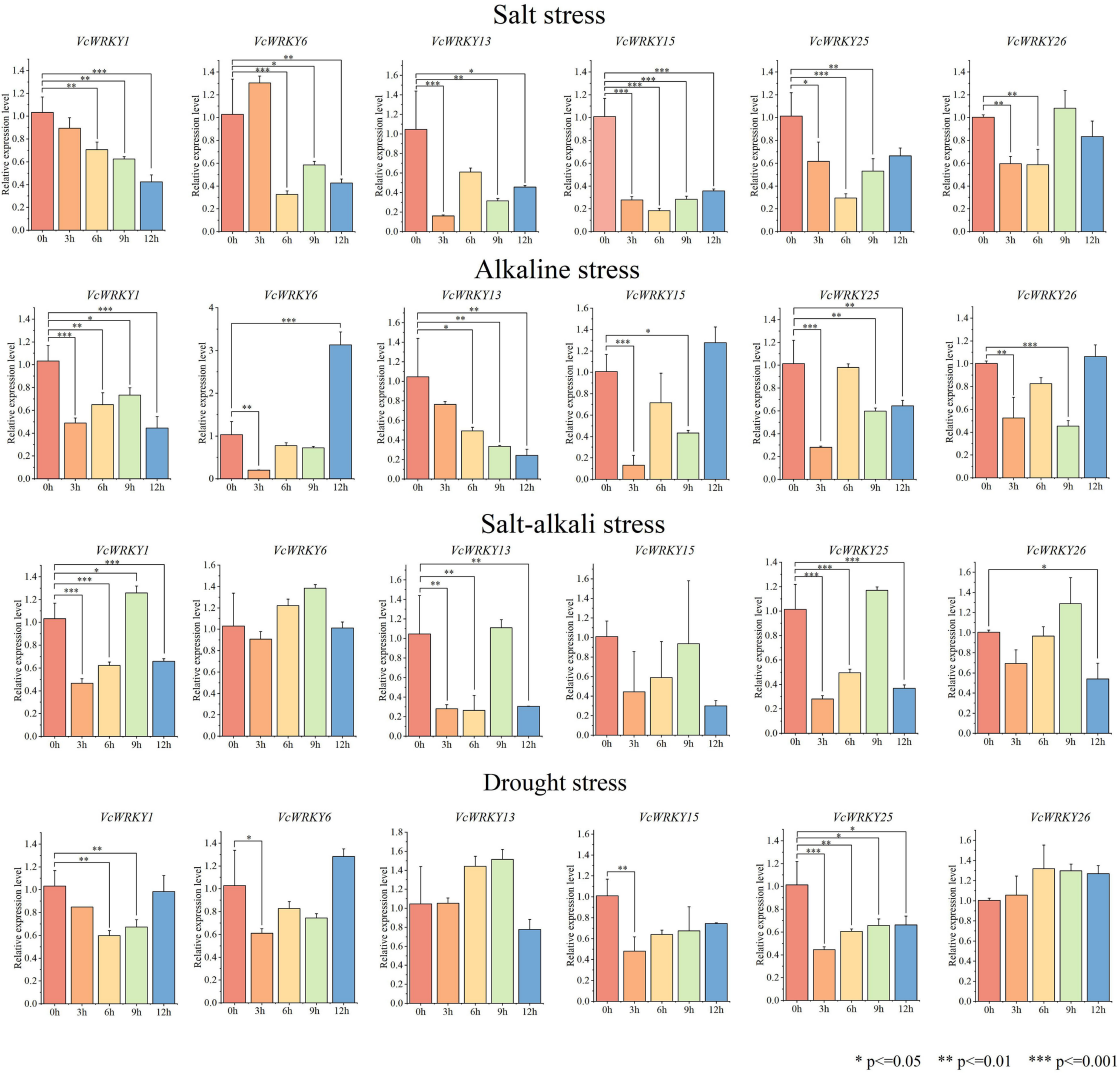
Expression patterns of *VcWRKY* genes in blueberry roots after four stress treatments. The ^*^ indicates significant differences by t-test (*p≤0.05, **p≤0.01, ***p≤0.001).

Under different stress treatments, the expression patterns of *VcWRKY* genes in roots were generally weaker than those in leaves (**Figure 8**). Specifically, when roots were subjected to salt stress, the expression levels of all other genes decreased, except *VcWRKY6*. *VcWRKY6* showed a peak in expression at 3 h, followed by a rapid decrease, indicating that it might be involved in the early response of blueberry roots to salinity stress. Similarly, under alkali stress, the expression levels of *VcWRKY6/15/26* peaked in roots after 12 h. *VcWRKY6* showed a three-fold increase in expression. Additionally, the expression levels of *VcWRKY1/25* generally decreased in roots, remaining below pre-treatment levels despite a slight increase. Except for *VcWRKY15*, the other five genes showed somewhat elevated expression levels under salt-alkali stress, peaking at 9 h. In response to drought stress, *VcWRKY1/6* showed similar response patterns, peaking after 12 h. Under drought stress, most genes did not show significant differences in expression levels. The expression levels of *VcWRKY13*/*26* were higher at 9 h and 6 h, respectively. Similarly, *VcWRKY15*/*25* showed gradually increasing expression levels during drought stress, although it remained below than pre-treatment levels.

### 3.11 Subcellular localization of VcWRKY proteins

**Figure 9:**
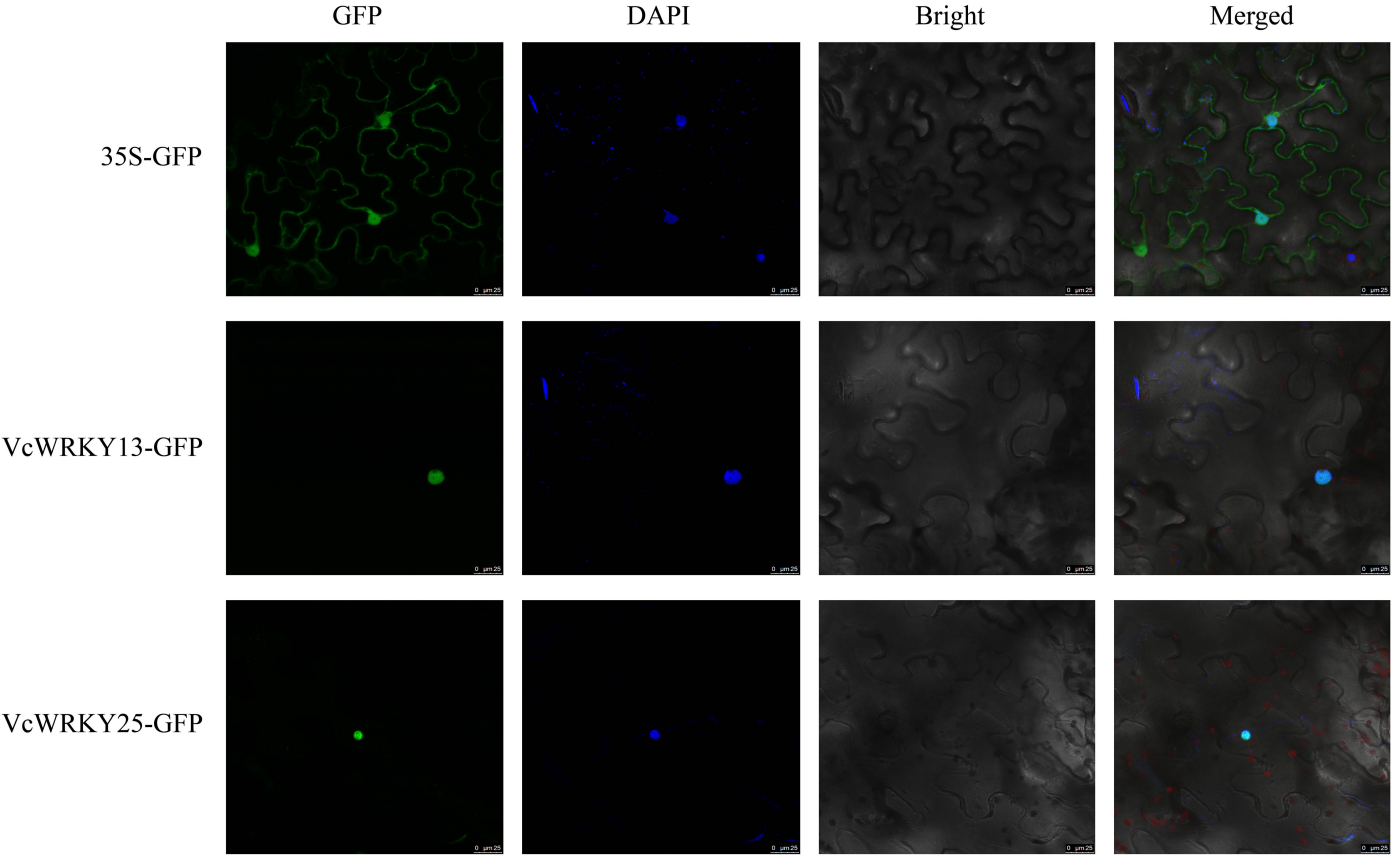
Subcellular localization of VcWRKY proteins in *N. benthamiana* leaves.

To further identify the localization of VcWRKY13 and VcWRKY25 in the cell, a transient expression assay was performed by introducing these two genes into the 35S-pGDG vector and transforming the recombinant vectors into *N. benthamiana* leaves. The results showed that VcWRKY13-GFP and VcWRKY15-GFP fusion protein were localized to the nucleus with 4’,6-diamidino-2-phenylindole (DAPI) staining. In contrast, the empty vector containing GFP was distributed across various organelles in *N. benthamiana* leaves (**Figure 9**). The results were consistent with the prediction made through bioinformatics analysis.

## 4 Discussion

Numerous environmental challenges, such as high salinity, dryness, and high temperatures, affect the growth and productivity of plants (Zhu, 2016). To mitigate these stresses, plants have developed complex regulatory mechanisms that involve various TFs (Sharif et al., 2021). Among these, *WRKY* genes are widely distributed and play a critical role in regulating stress responses (Baillo et al., 2019). As a key regulator in plants’ immune responses to various abiotic stresses, *WRKY* has been identified in several fruit trees, such as grape, blackberry, sweet orange, kiwifruit, and bilberry (Guo et al., 2014; Da Silva et al., 2017; Jing and Liu, 2018; Wu et al., 2022; Felipez et al., 2023). Many genes associated with abiotic stress tolerance in blueberries, including *VcLON1, VcLON2, VcBBX, VcMYB4a, VcDof*, and *VabZIP12*, have been identified (Chen et al., 2017; Li et al., 2022; Liu et al., 2023; Zhong et al., 2019; Zhang et al., 2020; Qu et al., 2022). It is yet unknown which specific *WRKY* genes in blueberries react to abiotic stress. Therefore, the main goals of our research were to identify the *WRKY* gene family in blueberries and to analyze the expression level under abiotic stresses. We identified 57 *VcWRKY* genes, all sharing similar structural domains, including a conserved WRKYGQK domain at the N-terminus and a zinc finger structure at the C-terminus in the same subgroup. In addition to WRKYGQK domain and the zinc finger structure, *VcWRKY7* and *VcWRKY20* showed additional *bZIP* and zinc cluster structures. Research by Qu et al. (2022) revealed that *VabZIP12* increases the levels of SOD, POD, and CAT in transgenic *Arabidopsis*, thereby improving resistance to salt stress. Thus, it will be possible to investigate the potential functions of *VcWRKY7* and *VcWRKY20* in salt stress resistance in future research.

Gene replication modes, including whole genome duplication (WGD), tandem duplication, segmental duplication, and transposon-mediated duplication, are significant mechanisms in eukaryotic genome evolution (Panchy et al., 2016). Tandem and segmental duplication events are the primary causes of the expansion of the *WRKY* gene family (Rushton et al., 2010). Within the *WRKY* gene family in pineapple, seven tandem repeat gene pairs and seventeen fragment duplication events have been identified (Xie et al., 2018). In Pitaya, eleven tandem duplication events were observed, involving two pairs of segmentally duplicated genes and 26 *HuWRKY* genes (Chen et al., 2022). According to Jia et al. (2022), 71 *WRKY* genes were distributed across 12 synteny blocks, comprising 31 segmental duplications and seven pairs of tandem duplications in bananas. 19 colinearity pairs involving 30 *PeWRKY* genes were identified in passion fruit (Ma et al., 2024). It is hypothesized that tandem and fragment replication events contribute to the expansion of the *VcWRKY* gene family. In blueberry, twelve synteny segment pairs were identified, and the *WRKY* genes showed tandem repeat occurrences within the same subfamily. Group I contained six pairs of *VcWRKY* genes, while group IIc had two pairs of duplicates. Groups IIa, IId, IIe, and III each had one pair of duplicates. A synteny map of *VcWRKYs* with monocotyledon and dicotyledon was also created, and it revealed 131 synteny pairs with *Actinidia* and 25 pairs with *Oryza.* Given the fewer synteny gene pairs between monocotyledons and blueberries, it is likely that these gene pairs formed after the divergence of dicotyledons and monocotyledons.

Gene functions can be inferred by comparing the homology of *VcWRKY* genes with those from other species. In *Arabidopsis*, *AtWRKY25/26/33* were known to participate in disease resistance, low-temperature resistance, and salt resistance, with their promoters containing various *cis*-elements responsive to abiotic stress (Fu and Yu, 2010). The phylogenetic analysis revealed that *VcWRKY13* shared a branch with *AtWRKY25/26/33*, indicating possible functional similarities. Furthermore, the expression of *VcWRKY13* significantly increased under salt stress, suggesting a potential role in salt resistance. According to Vanderauwera et al. (2012), *AtWRKY7* in *Arabidopsis* regulated the development of leaves, stems, and petals and contribute to drought and heat resistance. The target genes of *AtWRKY7*, RD26 and HSFA7a, were significantly upregulated in response to drought and high temperatures (Bai, 2021). *VcWRKY25*, a homolog of *AtWRKY7*, showed strong reactions to various abiotic stresses, indicating its role in blueberry.

As trans-acting regulators, *WRKY* genes can specifically identify and bind to various *cis*-elements in target genes, thereby controlling their expression either directly or indirectly (Xu et al., 2018). A multitude of *cis*-acting elements had been discovered in the upstream 2 kb region of the *VcWRKY* genes, which can be divided into four groups: phytohormone responsive, development related, stress related, and light responsive. Among the 57 *VcWRKY* genes, 202 CGTCA motifs, 171 ABRE elements, 145 ARE motifs, 49 LTR sequences, and 33 MBS motifs were identified. In *VcWRKY* genes, the majority of *cis*-acting regulatory elements were associated with MeJA and abscisic acid responsiveness. This study used the cold-resistant cultivar ‘Northland’, whose cold resistance may have been attributed to the abundance of MeJA-responsive elements in the promoters of the *VcWRKY* genes. Further investigations will be necessary to elucidate the mechanisms by which MeJA regulates *VcWRKYs* in response to stress. Additionally, a considerable number of MYB and MYC recognition sites were identified in the *VcWRKY* promoters. Zhang et al. (2020) found that the *VcMYB4a* was downregulated under drought, salt, and cold stresses and that overexpression of *VcMYB4a* in the callus of blueberries heightened sensitivity. Furthermore, Wang et al. (2022) found that the major transcription factors *VcABR1, VcABF2, VcMYB108*, and *VcMYB93* were probably implicated in drought stress responses in leaves and roots of ‘Bluecrop’. The promoter of *VcWRKYs* exhibited a large number of MYB recognition sites, suggesting significant interaction between *VcWRKYs* and *VcMYBs*. Future research can focus on examining the interactions between these two important transcription factor families.

The regulatory roles of *WRKY* genes in plant stress responses have been the subject of numerous investigations. For instance, 12 *AcWRKYs* in kiwifruit response to salt and drought stress (Jing and Liu, 2018). Similarly, *VvWRKY13/45/71* in grapes showed significant responses to low temperature and salt stress, with peaking at 6 h after treatment (Hou et al., 2013). In tomato, under salt stress, *SlWRKY13/31/50/62/63* were up-regulated, with peak expression levels early in the stress response. *SlWRKY63* expression increased significantly, reaching 500-fold at 3 h under drought stress (Zhang, 2017). In this study, *VcWRKYs* exhibited variable degrees of responses to salt stress, alkali stress, salt-alkali stress, and drought stress. In response to salt stress, *VcWRKY13/25* showed a significant upregulation, with increases of 48-fold and 45-fold, respectively. Furthermore, some genes displayed contrasting expression levels in various organs. For example, under salt stress, *VcWRKY25* was upregulation in leaves but downregulated in roots. The response of *VcWRKY1* to drought stress was more noticeable in roots than in leaves. Similarly, in upland cotton under salt stress, the expression levels of *GhWRKY41* in roots and stems peaked at 6 h and 48 h, respectively, while in leaves, it declined (Su et al., 2016). Further research will be needed to elucidate the precise regulatory mechanisms of *VcWRKY* genes in response to abiotic stress.

## 5 Conclusion

In blueberries, 57 *VcWRKY* genes were identified, distributed across 32 chromosomes and divided into five subgroups. The study analyzed the physicochemical properties, phylogeny, gene structure, conserved motifs, *cis*-acting elements, and synteny of these WRKY proteins. The VcWRKY proteins were localized in the nucleus and exhibited the conserved WRKY heptapeptide structure and zinc-finger motif. Organ-specific expression patterns of *VcWRKYs* were determined through RT-qPCR analysis, *VcWRKY9, VcWRKY10*, and *VcWRKY27* showed higher expression levels across 12 distinct blueberry tissues. Significantly, *VcWRKY13* and *VcWRKY25* showed strong reactions to salt stress, alkali stress, saline-alkali stress, and drought stress, indicating their potential roles in stress resistance. This finding provides insights into the fundamental characteristics of the *VcWRKY* gene family, offering a foundation for identification of stress-related genes for the development of blueberry cultivars.

## Data Availability

The datasets presented in this study can be found in online repositories. The names of the repository/repositories and accession number(s) can be found in the article/**Supplementary Material**.
